# A Bio-Inspired Memory Model Embedded with a Causality Reasoning Function for Structural Fault Location

**DOI:** 10.1371/journal.pone.0120080

**Published:** 2015-03-23

**Authors:** Wei Zheng, Chunxian Wu

**Affiliations:** Key Laboratory for Optoelectronic Technology and System of the Education Ministry of China, College of Optoelectronic Engineering, Chongqing University, Chongqing, China; Southwest University, CHINA

## Abstract

Structural health monitoring (SHM) is challenged by massive data storage pressure and structural fault location. In response to these issues, a bio-inspired memory model that is embedded with a causality reasoning function is proposed for fault location. First, the SHM data for processing are divided into three temporal memory areas to control data volume reasonably. Second, the inherent potential of the causal relationships in structural state monitoring is mined. Causality and dependence indices are also proposed to establish the mechanism of quantitative description of the reason and result events. Third, a mechanism of causality reasoning is developed for the reason and result events to locate faults in a SHM system. Finally, a deformation experiment conducted on a steel spring plate demonstrates that the proposed model can be applied to real-time acquisition, compact data storage, and system fault location in a SHM system. Moreover, the model is compared with some typical methods based on an experimental benchmark dataset.

## Introduction

Monitoring tasks are increasingly complex. Hence, structural health monitoring (SHM) technology has entered a stage of intelligent development in which individual data, signal, and knowledge processing procedures are presently integrated. Knowledge-based techniques are usually combined with several intelligent methods to identify structural faults[1–3]. However, some notable problems in industrial applications must be resolved urgently.

First, massive data storage pressure is an outstanding issue in long-term SHM systems. To save on storage costs, sampling frequency is often reduced. However, strong external disturbances such as earthquakes may occur in the interval between samplings, which may last a few minutes. These disturbances may result in an unrecorded change in structural state [4]. Data compression techniques can also be used to save on storage cost, including methods based on fast Fourier transform, wavelet transform, and model-based compression [5–7]. However, complex compression algorithms lengthen the response time of the system, and processing the large amounts of corresponding data inevitably enhances computational difficulties [8]. Nonetheless, the process of instantly capturing structural changes while controlling data volume reasonably remains a challenge.

Second, a structural fault usually deteriorates gradually and extends from the focus to the surface. This process is difficult to monitor using a structural mathematical model when the effect of structural degradation and environmental changes is considered [9]. Recent studies have measured a single parameter in multiple channels, such as the monitoring of stress, strain, and pressure [10,11]. The consideration of a combined effect strongly complicates fault analysis given the individual differences in the monitored signals.

Thus, the current study establishes a bio-inspired memory model embedded with causality reasoning function for structural fault location in consideration of how the human memory works and of the causal relationship in the structural fault process. This approach does not require a precise mathematical structural model and can be used for real-time acquisition, compact data storage, and system fault location in a SHM system.

## Related Works

From a psychological perspective, memory is the ability of an organism to store, retain, and subsequently retrieve information. In 1968, Atkinson and Shiffrin proposed a model of human memory that posits three distinct memory stores: sensory, short-term, and long-term memory[12]. The study conducted by Ruff shows that neural processing in the sensory cortical areas can be biased toward behaviorally relevant stimuli and toward thoughts generated by feedback projections from the frontal and parietal brain areas [13]. Nee and Jonides reviewed the evidence that short-term memory is an amalgamation of three qualitatively distinct states [14]. Widrow and Aragon reported that memory and pattern recognition are interrelated and justified why long-term memory is stored in DNA or RNA[15]. Bacca, Salvi, and Cufi proposed a system for long-term mapping and localization based on the introductory concepts of short-term and long-term memory [16]. Wang and Qi presented a memory-based cognitive model for visual information processing as inspired by human perception of an environment [17]. Song, Weng, and Lebby developed a dynamic model for the flapping motion control of micro air vehicles under a control scheme inspired by human memory [18].

Moreover, structural faults develop progressively with the inherent potential of causal relationships [19,20]. This condition guides the authors in mining the causal relationship of the monitored signals for system fault location.

Causal relationships exist in various fields. Furthermore, several reason events occur in different timing sections; these events result in either the onset or the conclusion of result events [21,22]. The present techniques that evaluate causal information are mainly based on probability theories, such as Bayesian network theory. A Bayesian net is a directed acyclic graph that can be used to indicate the probability of the causal relationships among events. This theory is widely used in fields such as fault diagnosis and pathology inference [23,24]. Several multi-technical fusion methods have also been established for causal information assessment. For example, Sharda and Banerjee combined the Bayesian net with a genetic algorithm in a robust design [25]. Baldwin and Di Tomaso [26] reduced the complexity of the automatic learning of a Bayesian net from the data using a fuzzy set.

The aforementioned methods generally consider conditional probability to be the modelling foundation, and they lack temporal information regarding an event. Therefore, Wen et al. [27] identified the correlation of the time series between cause and effect events. Karimi and Hamiton [28] integrated temporal information into a decision tree of causal relation. Arnold, Liu, and Abe [29] established a temporal causal model through graphical modeling based on the concept of “Granger causality”. Mosterman and Biswas [30] developed temporal causal graphs to extend the traditional causal constraints by including the temporal constraints that are important in the analysis of dynamic systems. However, causality is insufficiently described quantitatively because the application of causality to locate faults in a SHM system is novel.

## Model

Most scientists agree that human memory can be described as a set of stores into which information is “placed”. A set of processes then acts on these stores. A very simple model may contain three different stores: sensory information store (SIS), short-term store (STS), and long-term store (LTS). It may also include three processes: encoding (inputting information into a store), maintenance (keeping this information “alive”), and retrieval (determining encoded information) [31].

Much information from the outside world is filtered through our sight, hearing, smell, taste, and touch sensors. We store this sensory information in the cortical areas of the brain. The data that catch our attention or are urgently needed are moved to the short-term memory. Sensory memory corresponds to that generated in approximately the initial 200 milliseconds–500 milliseconds after an item is perceived. Short-term store (STS) enables one to recall a memory that ranges from several seconds to a maximum of a minute without rehearsal. However, its capacity is highly limited. There are three ways in which one can forget information in the STS: decay, displacement and interference. By contrast, long-term memory can store much larger quantities of information for a potentially unlimited duration (occasionally, an entire lifespan).Still though, we can forget information through decay (as in short-term forgetting) and interference from other memories [32–34].

**Fig 1:**
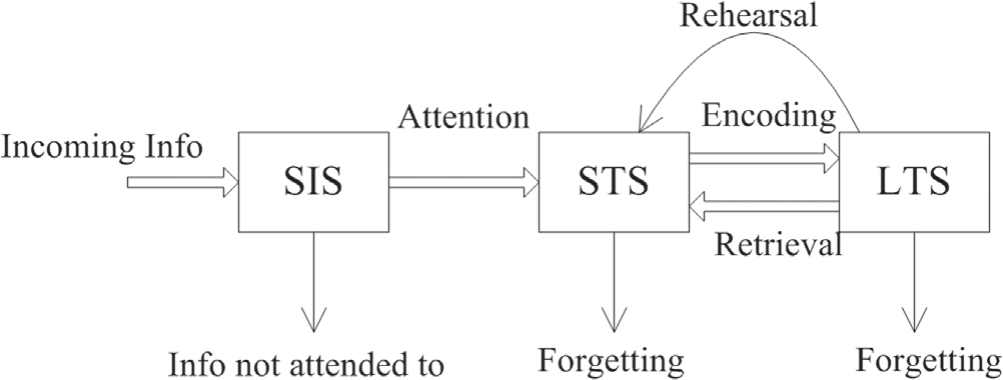
Working process of human memory.

Fig. 1 shows the working process of the human memory[35].

This biological system filters out useful information from various sources, such as sight, hearing, and smell, while discarding much useless data. The process is similar to filtering, which effectively reduces data volume.

**Fig 2:**
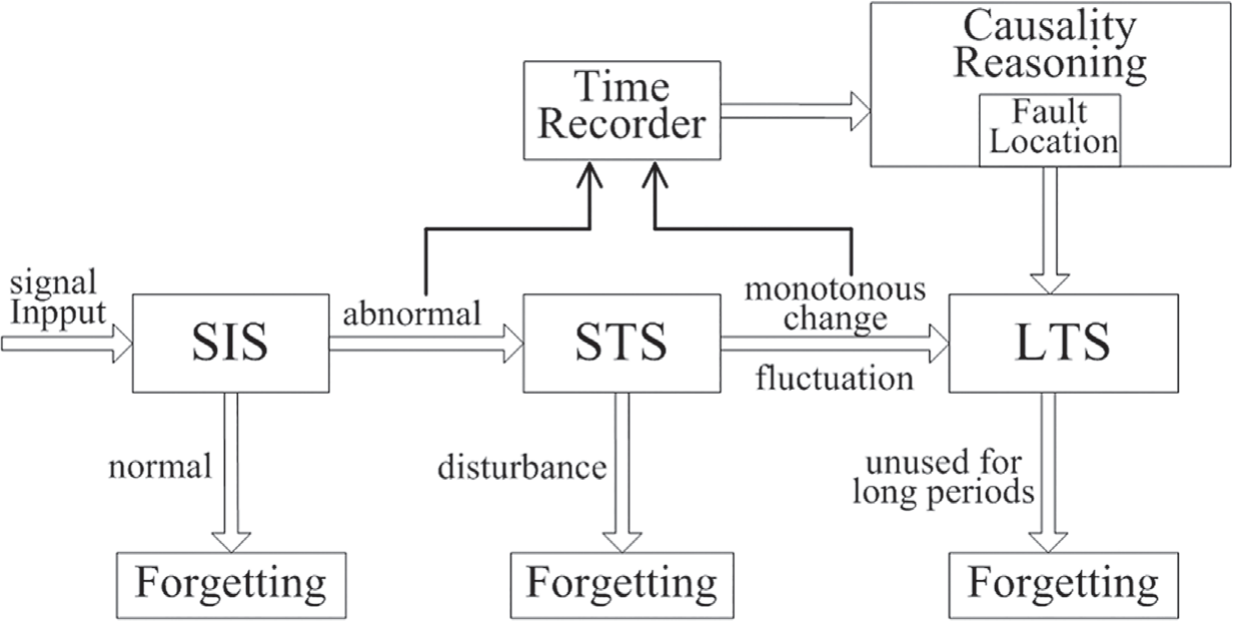
Architecture of BMCRFE model.

Inspired by the features of human memory, this study establishes the bio-inspired memory model embedded with causality reasoning function for structural fault location as shown in Fig. 2. This study simulates the behavior of human memory based on knowledge regarding psychology and behavioral science. Human memory differs from artificial neural networks such as dendrites and axons, which imitate the physical structure of the human nervous system. Therefore, the study of the human memory does not require sophisticated physiological knowledge. The inspired memory model is primarily used to compress the volume of data. Furthermore, a causal inference function is integration into the LTS for structural fault location.

This study divides the SHM data to be processed into three temporal areas: SIS, STS, and LTS. First, the acquired data enter the SIS, in which they are maintained for a few seconds. If the value of the information is within the normal range of the structural state, this information is instantly forgotten. Otherwise, the signal proceeds to the STS for further observation. The onset and end times of the abnormal signals are recorded in this area. Much of the uninteresting data collected by the SHM system is discarded in the process, thus alleviating data storage pressure considerably.

The process can be described as follows:

Let *x*_*i*_ represent the structural property monitored by sensor *i*. y_*i*_ denotes the output of sensor *i*; *t*_*j*_ corresponds to the current monitoring time; Δ*T*_*SIS*_ indicates the time width of the SIS area; and *θ*_*i*_ represents the fluctuation limit. If the original normal value is *y*_*i_normal*_, then we obtain

$$y_{i}(t_{j})=f(x_{i},t_{j}),\;0\leq t_{j}\leq \Delta T_{SIS},$$ (1)

where *f*(*x*_*i*_, *t*_*j*_) is the function of sensory output.

1. If |*y*_*i*_(*t*_*j*_) – *y*_*i_normal*_| < *θ*_*i*_, then *y*_*i*_(*t*_*j*_), *j* = *j* + 1 is disregarded;
2. If |*y*_*i*_(*t*_*j*_) – *y*_*i_normal*_| ≥ *θ*_*i*_, then *y*_*i*_(*t*_*j*_) and *t*_*j*_ are recorded. The following process is then applied for operation:
  If *t*_*j*_ ≤ Δ*T*_*SIS*_, then *j* = *j* + 1 should be observed further;
  If *t*_*j*_ > Δ*T*_*SIS*_, then the signal is switched to STS.

In STS, the signal from the SIS is monitored for either a few seconds or a few minutes, which is longer than the monitoring duration in the SIS. If the structural state fluctuates, the data related to the stable structural balance state are sent to the LTS. The SHM system detects serious damage to the structure if the signal values exceed the normal range and either increase or decrease monotonously. The onset and end times of the monotonous change are then recorded. The system determines SIS disturbance if the signal values return to the normal range and abnormal monitoring data are no longer observed. The structural state stabilizes after further monitoring in the STS. Thus, monitoring data need not be sent to the LTS, and the STS disregards the disturbance monitoring data.

The working principle of the short-term memory area can be described as follows:

Suppose the signal stream is *y*_*i*_(*t*_*j*_), Δ*T*_*SIS*_ < *t*_*j*_ ≤ Δ*T*_*SIS*_ + Δ*T*_*STS*_.

1. If |*y*_*i*_(*t*_*j*_) – *y*_*i_normal*_| < *θ*_*i*_, then *y*_*i*_(*t*_*j*_) is recorded and *j* = *j* + 1 should be observed further.
2. If |*y*_*i*_(*t*_*j*_) – *y*_*i_normal*_| ≥ *θ*_*i*_, then *y*_*i*_(*t*_*j*_) is recorded and *j* = *j* + 1 should be examined further.

If branch 1) is always followed in the time interval [Δ*T*_*SIS*_, Δ*T*_*SIS*_ + Δ*T*_*STS*_], the recorded data in the STS can be forgotten because this time interval suggests that abnormal data are no longer generated.

Branch 2) evaluates this situation further as follows:

If *y*_*i*_(*t*_*j*_) ≥ *y*_*i*_(*t*_*j* −1_), *j* = *h*, *h* + 1, ……*h* + *f* or *y*_*i*_(*t*_*j*_) ≤ *y*_*i*_(*t*_*j−1*_), *j* = *h*, *h* + 1, ……*h* + *f*, then an alarm signal is emitted immediately because these expressions indicate that the signal values exceed the normal range and either increase or decrease monotonously *f* times from time *t*_*h*_. The onset and end times of this monotonous change are then recorded, and the monitoring information is then sent to the LTS.

If the structural state fluctuates and *t*_*j*_ > Δ*T*_*SIS*_ + Δ*T*_*STS*_, then the new balance location of the structure can be determined. The information is then sent to the LTS.

LTS is a large-capacity storage area in which information can be preserved for a long time. Long-term memory stores the changing parameters of the structural state and the results of structural fault identification derived from the causality reasoning unit. Additional data are stored in the LTS to provide information for structural state analysis. The LTS data that remain unused for long periods of time are forgotten to control data volume reasonably.

## Causality Reasoning

### Part 1. Analysis of causal relations

Causal relationships can be described as several reason events occurring in different timing sections that result in either the onset or the conclusion of result events.

When *T* represents the time set for event set *D*, *T*|*D* = [*TB*, *TE*], where *TB* and *TE* are the onset and end times of *D*, respectively.

**Fig 3:**
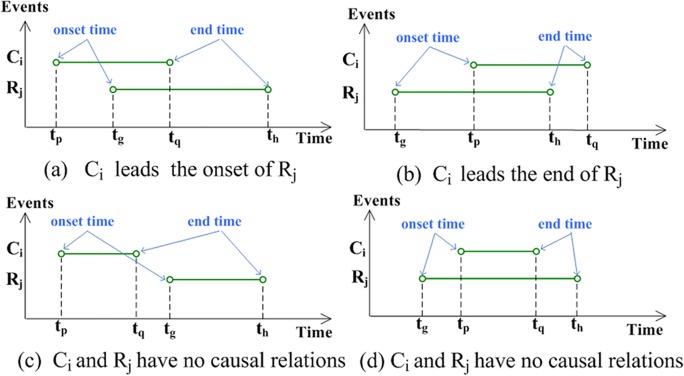
Typical relations between Ci and Rj.

Suppose the reason event set is *C* = {*C*_1_, …, *C*_*m*_} and the result event set is *R* = {*R*_1_, …, *R*_*n*_}:

*C*_*i*_ and *R*_*j*_ are related in several ways given *T*|*C*_*i*_ = [*t*_*p*_, *t*_*q*_], *T*|*R*_*j*_ = [*t*_*g*_, *t*_*h*_] (*i* = 1, 2, …, *m*; *j* = 1, 2, …, *n*; 1 ≤ *p*, *q*, *g*, *h* ≤ *k*). The typical relations are presented in Fig. 3.

If events *C*_*i*_ and *R*_*j*_ are causally related, event *C*_*i*_ should lead to either the onset or the end of event *R*_*j*_ (Fig. 3[A] and 3[B]). If events *C*_*i*_ and *R*_*j*_ do not intersect in the time domain, then they should not be causally related (Fig. 3[C]). Although events *C*_*i*_ and *R*_*j*_ intersect in the time domain, they are not related in this manner as well (Fig. 3[D]).

### Part 2. Quantitative calculation

Different disturbances are attributed to various structural faults in a SHM system [36,37]. Some structural faults originate from the effect of the onset of a reason event, whereas others are induced by the cumulative influence of the reason event. For example, an earthquake is a strong external disturbance to a structure, and its effect is instant. By contrast, a vehicle load places pressure on a road, but its effect accumulates over time. The present paper proposes a quantitative calculation method for causality based on the onset and cumulative influences of the reason events.

The onset effect of events *C*_*i*_ to *R*_*j*_ in Fig. 3(A) can be expressed as

$$k_{1}/[t_{g}(R_{j})-t_{p}(C_{i})]$$ (2)

where *k*_1_ is the onset effect coefficient. 0 < *k*_1_ ≤ 1.

Given *C*_*i*_ ∈ *C*, (i = 1, 2, …m), a small value of *t*_*g*_(*R*_*j*_) − *t*_*p*_(*C*_*i*_) indicates that the onset effect of event *C*_*i*_ on event *R*_*j*_ is strong.

The cumulative influence of event *C*_*i*_ on event *R*_*j*_ in Fig. 3(A) can be expressed as

$$k_{2}[t_{q}(C_{i})-t_{g}(R_{j})]/{\left[t_{h}(R_{j})-t_{g}(R_{j})\right]}^{2}$$ (3)

where *k*_2_ is the cumulative effect coefficient. 0 < *k*_2_ ≤ 1.

A high value of [*t*_*q*_(*C*_*i*_) − *t*_*g*_(*R*_*j*_)/[*t*_*h*_(*R*_*j*_) − *t*_*g*_(*R*_*j*_)]^2^ suggests that event *C*_*i*_ has a strong cumulative influence on event *R*_*j*_ when *C*_*i*_ ∈ *C*, (i = 1, 2, …m).

Hence, the present study defines a causality degree as follows:

**Definition 1.** ∀*T*|*C*, *T*| *R*, *C*_*i*_ (*i* = 1, 2, …*m*) is assigned as the row vector, and *R*_*j*_(*j* = 1, 2, …*n*) is the column vector. The 2D matrix *σ*_*m×n*_ is then established. The value of *σ*_*ij*_ is the causality index and reflects the causality strengths of *C*_*i*_ and *R*_*j*_.

Given the causality calculation method displayed in Fig. 3, the computation formula for *σ*_*ij*_ can be expressed as follows:

$$\begin{array}{l} \exists T|C_{i}=[t_{p},t_{q}],T|R_{j}=[t_{g},t_{h}],\;\left(i=1,2,\ldots ,m;\;j=1,2,\ldots ,n;\;1\leq p,q,g,h\leq k\right)\\ \sigma_{ij}=\left\{ \begin{array}{l} \begin{array}{lll} k_{2}\frac{t_{q}(C_{i})-t_{g}(R_{j})}{{\left[t_{h}(R_{j})-t_{g}(R_{j})\right]}^{2}}\text{ ,} & {\text{t}}_{p}({\text{C}}_{\text{i}})=t_{g}(R_{j}) & \\ k_{1}\frac{1}{t_{g}(R_{j})-t_{p}(C_{i})}\text{ +}k_{2}\frac{t_{q}(C_{i})-t_{g}(R_{j})}{{\left[t_{h}(R_{j})-t_{g}(R_{j})\right]}^{2}}\text{ ,} & t_{p}(C_{i})<t_{g}(R_{j})\leq t_{q}(C_{i}) & \\ & & 0<k_{1},k_{2}\leq 1\\ k_{1}\frac{1}{t_{h}(R_{j})-t_{p}(C_{i})}\text{+}k_{2}\frac{t_{h}(R_{j})-t_{p}(C_{i})}{{\left[t_{h}(R_{j})-t_{g}(R_{j})\right]}^{2}}\text{ ,} & t_{g}(R_{j})<t_{p}(C_{i})<t_{h}(R_{j})\leq t_{q}(C_{i}) & \end{array}\\ \;0\;\;\;\;\;\;\;\;\;\;\;\;\;\;\;\;,\;\text{other cases} \end{array}\right. \end{array}$$ (4)

Formula (4) quantitatively calculates the causality strengths of the reason and result events.

Another index known as the dependence index is defined to evaluate causal knowledge and to locate system faults.

**Definition 2.** The onset or end of *R*_*j*_ relies on the cooperation of all of the elements in set C in ∃*R*_*j*_ ∊ *R*(*j* = 1, 2, …*n*). The dependence level of *R*_*j*_ on *C*_*i*_ is called the dependence index *DEP*_*i*_(*R*_*j*_) given *C*_*i*_ ∈ *C*(*i* = 1, 2, …*m*).

*DEP*_*i*_(*R*_*j*_) can be calculated in combination with the probability method using the following formula:

$$DEP_{i}\left(R_{j}\right)=\frac{W_{d}(C_{i},R_{j})*\sigma_{ij}}{\sqrt{\sum_{i=1}^{m}{\left[W_{d}(C_{i},R_{j})*\sigma_{ij}\right]}^{2}}}$$ (5)

*W*_*d*_(*C*_*i*_, *R*_*j*_) is the strength of the dependence connection strength between *C*_*i*_ and *R*_*j*_. It is designed to provide probabilistic reinforcement in our study. If *DEP*_*i*_(*R*_*j*_) (*i* = 1, 2, …*m*) is calculated individually and the results are arranged in descending order, then the event *C*_*i*_ that displays a high dependence index value is highly likely to be the reason event of *R*_*j*_. Thus, system faults can be located based on knowledge reasoning from the result event to the reason event.

For instance, reason event *C*_*i*_ can be denoted as the monitoring information collected by the numerous sensors of a complex structural state monitoring net, whereas overall structural performance can be taken as result event *R*_*j*_, including collapse, distortion, and fracture. An event *C*_*i*_ with a high dependence index value is the most likely reason event for a given result event (structural state output). Therefore, the position of the corresponding sensor for this event is emphasized in system fault location.

To simplify the original values of *W*_*d*_(*C*_*i*_, *R*_*j*_), we set these values to $\frac{1}{m}$ in the present study. Nonetheless, we subsequently designed a rule to dynamically adjust these values. Thus, parameters are self-renewable in the model. The rule is explained as follows:

Following the calculation of *DEP*_*i*_(*R*_*j*_) (*i* = 1, 2, …*m*; *j* = 1, 2, …*n*), the new value of *W*_*d*_(*C*_*i*_, *R*_*j*_) can be regulated dynamically using the following formula:

$$W_{d}(C_{i},R_{j})=\frac{1}{m}+KD_{ij}*\nu_{d}\;\left(i=1,2,\ldots m;j=1,2,\ldots n\right)$$ (6)

where $KD_{ij}=\frac{DEP_{i}^{*}(R_{j})}{\sum_{i=1}^{m}DEP_{i}^{*}(R_{j})}.\;DEP_{i}^{*}\left(R_{j}\right)$ is the previous value of *DEP*_*i*_(*R*_*j*_) and *v*_*d*_ is a slight increment.

A high dependence index value enhances dependence connection strength, and the value of *W*_*d*_(*C*_*i*_, *R*_*j*_) increases accordingly to prepare for the next causality evaluation.

### Part 3. Causality reasoning for system fault location

**Fig 4:**
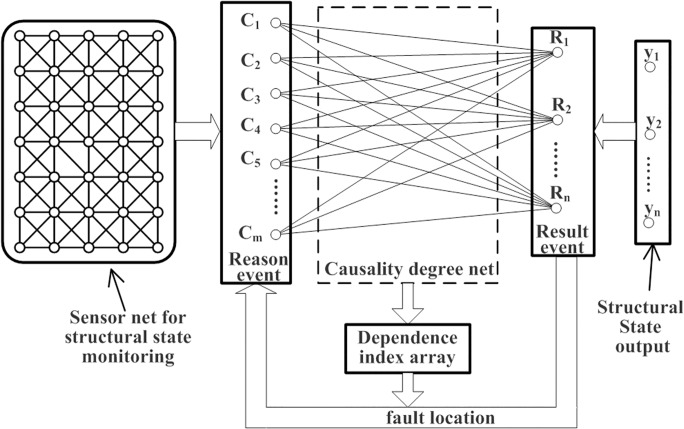
Causality reasoning for system fault location.

Based on the aforementioned methods, a causality reasoning mechanism for system fault location is proposed as follows (Fig. 4):

1. The sensor net for structural state monitoring uses numerous sensors. Changes in the sensor signal are captured in real time. Subsequently, the onset and end times of the abnormal signals are recorded, then the sensor net containing the temporal information is mapped to reason event set *C* = {*C*_1_, …, *C*_*m*_}.
2. Meanwhile, the onset and end times of the output of the structural fault state are recorded. The structural states include the comprehensive evaluation results obtained from the sensors. The structural state output with the temporal information is then mapped to result event set *R* = {*R*_1_, …, *R*_*n*_}.
3. *σ*_*ij*_ is calculated to establish the causality degree net.
4. Subsequently, the dependence index is dynamically calculated to evaluate the causal knowledge.
5. System faults are located by comparing the calculation results of dependence.
6. Finally, the dependence connection strength *W*_*d*_(*C*_*i*_, *R*_*j*_) is updated for the next causality evaluation.

The use of this model is appealing because it is relatively easy and simple to implement. It successfully avoids analyzing the differences among the numerous sensors in a complicated monitoring net while quantitatively calculating the inherent potential of the causal relationships between the monitoring processes and the results. Therefore, an intelligent fault location mechanism is established based on the causality reasoning for SHM.

## Results and Discussion

### Experiment System

**Fig 5:**
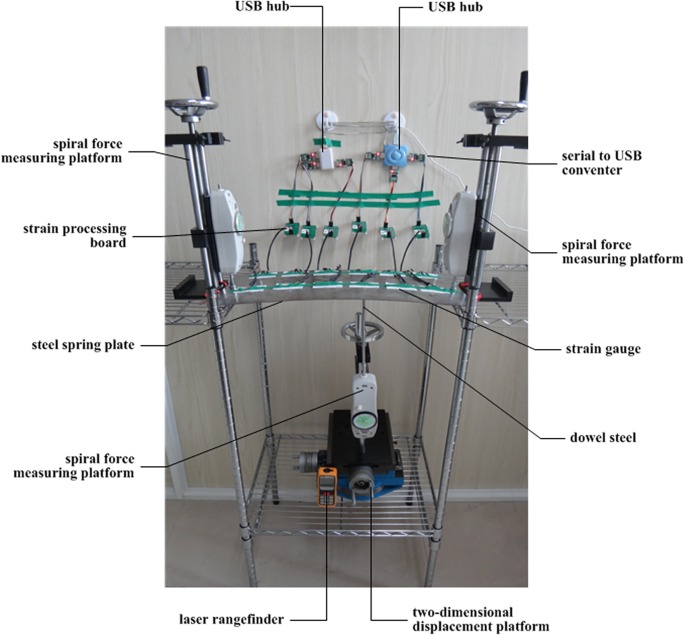
Experimental system.

An experimental system was established to demonstrate the validity of the proposed model and to concretely describe the application process of the model. The system is depicted in Fig. 5.

**Fig 6:**
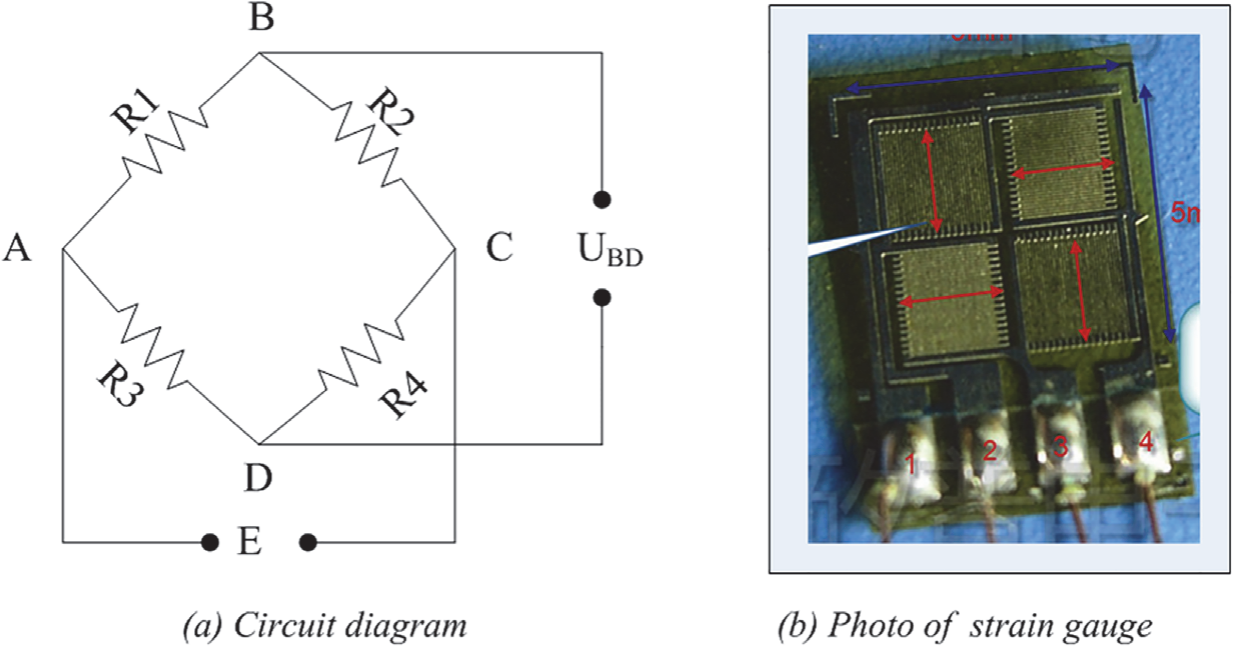
Circuit diagram and photo of each strain gauge.

This study monitors a steel spring plate on which 12 full-bridge, metal-foil strain gauges are affixed. Fig. 6 displays the images and circuit diagrams of each gauge, which are important in the experiment. The general Wheatstone bridge illustrated in Fig. 6(A) consists of four resistive arms. Moreover, an excitation voltage *E* is applied across the bridge. *U*_*BD*_ is the output voltage of the bridge.

Data are acquired using six strain processing boards with a serial communication interface. The monitoring data are sent to a serial to USB converter. Finally, the data is downloaded to a control computer through a USB hub. The 12 strain gauges are designed with connectors that can be easily installed or uninstalled such that the data from these sensors can be acquired alternately because only six independent strain boards are provided.

Furthermore, three spiral force-measuring platforms were used in the experiment. One platform is fixed onto a 2D displacement platform and is placed under the steel spring plate. A dowel steel is installed on the spiral force-measuring platform, which can be moved over 3D. If the torque handle on this platform is turned, torque stimulation is converted into pressure stimulation. This stimulation strains the steel spring plate through the dowel steel. The value of pressure stimulation can be measured using the dynamometer on the spiral force-measuring platform. Moreover, a laser rangefinder is also installed under the steel spring plate to measure its vertical deformation. The two other spiral force-measuring platforms are positioned on both sides of the steel spring plate. The upward pressures induced by the deformation of the steel spring plate are placed on the aforementioned platforms and are measured by the dynamometers.

As mentioned previously, the steel spring plate is strained by the dowel steel when the torque handle on the spiral force-measuring platform below the plate is turned. The force point is moved to a different area of the steel spring plate by controlling the 2D displacement platform. As a result, the steel spring plate is deformed in different ways in various locations and times.

**Fig 7:**
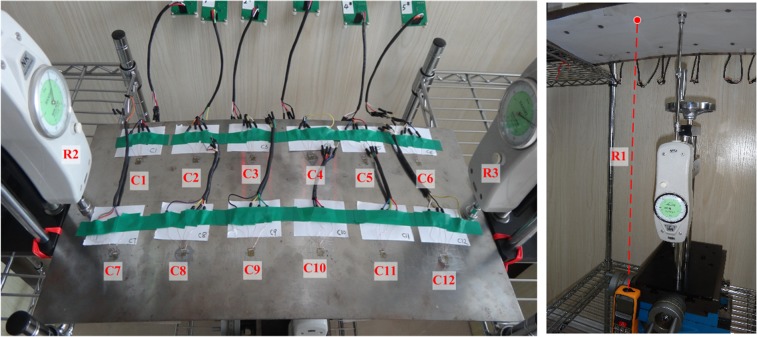
Corresponding relations between experiment system and causal information system.

Fig. 7 indicates the corresponding relations between the experimental and causal information systems.

The sensor information derived from the 12 strain gauges is regarded as reason event set *C* = {*C*_1_, …, *C*_12_}. The structural state outputs are the vertical deformation measured by the laser rangefinder and the upward pressures determined with the two spiral force-measuring platforms. Collectively, these outputs are considered result event set *R* = {*R*_1_, …, *R*_3_}.

### Data volume control

**Fig 8:**
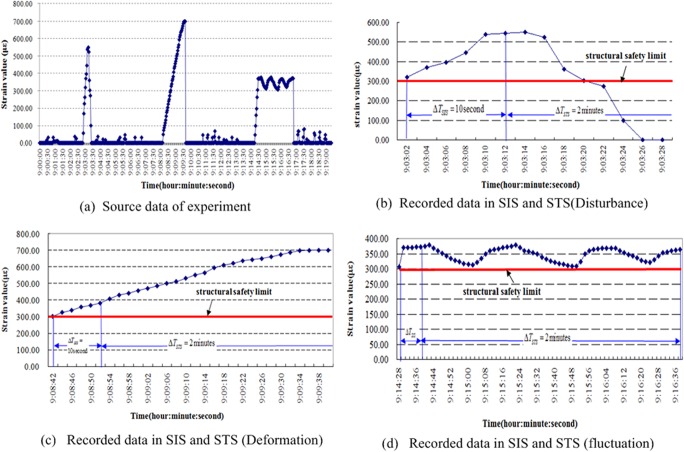
Data volume control experiment.

The figures cited in the following paragraphs are obtained from a group of data derived from numerous experiments within a specific period of time. The source data are the strain values measured by strain gauge C3, and the force point is located below it. In these experiments, disturbance, structural deformation, and structural fluctuation events were simulated over a period of 20 min. First, we recorded every datum acquired at a sample rate of 0.5 Hz as in tradition. The acquisition time was also recorded in the data acquisition software. Therefore, the stored data includes both the signal values and the acquisition time. The values derived from the strain gauges are shown in Fig. 8(A). με is the unit that corresponds to the Y-axe.

The source data were then inputted into the proposed model to verify their validity. Suppose that Δ*T*_*SIS*_ = 10 s and Δ*T*_*STS*_ = 2 min, and the initial limit of structural safety is θ_*i*_ = 300 με. Within time Δ*T*_*SIS*_, many normal data were disregarded in the SIS area. Rapid force was applied to the steel spring plate as a disturbance at approximately 9:02:54. The SIS captured the changes in the data at a new step time of Δ*T*_*SIS*_, which began at 9:03:02. Nonetheless, only abnormal data that exceeded the safety limit were recorded as presented in Fig. 8(B).

The same figure indicates that the signal entered the STS for further monitoring within the time Δ*T*_*STS*_. The signal values normalized, and abnormal data were no longer observed. The proposed model deduced transient disturbances to the structure; consequently, the STS disregards the disturbance monitoring data.

The steel spring plate was deformed slightly by the force applied at 9:08:42. The proposed model quickly captured the changing signal and recorded the data in the SIS and STS, as depicted in Fig. 8(C).

Fig. 8(D) indicates that if the signal values formed a fluctuating curve, then the structure can be balanced in a new location. The information is then sent to the LTS.

**Fig 9:**
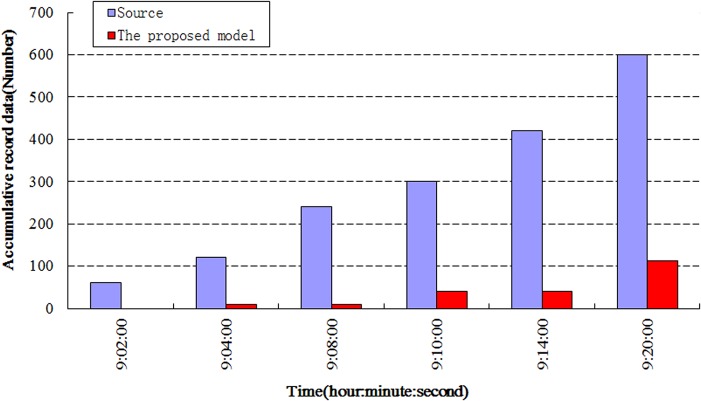
Comparative histogram of data storage.

The data storage space is well-managed when proposed technology is used. Fig. 9 exhibits the histogram of the comparison of the accumulated amount of recorded data with the amount of source data. The recorded data constitute only 18.6% of the source data in the experiment. Therefore, this result demonstrates that the proposed model efficiently alleviates data storage pressure on a computer.

### Causal information system

Fig. 7 illustrates that the sensor information is regarded as reason event set *C* = {*C*_1_, …, *C*_12_}. The structural state outputs are then considered result event set *R* = {*R*_1_, …, *R*_3_}.

**Fig 10:**
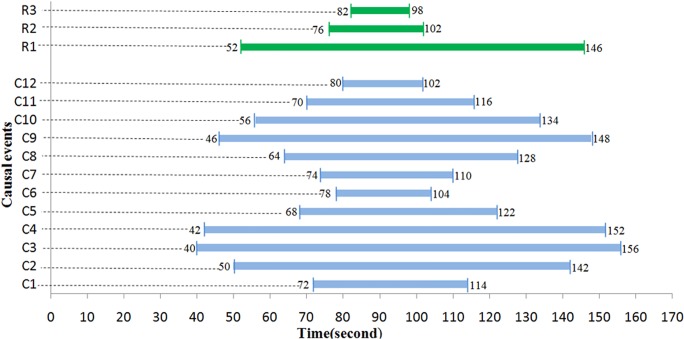
Temporal information acquired in experiment.

The steel spring plate is strained by turning the torque handle on the spiral-force measuring platform. The force point was located near C3, C4, and C9. The monitoring data regarding the reason and result events were processed using the proposed model. Moreover, the onset and end times of the abnormal signals were recorded. The resultant data volume was well-managed, and the temporal information was acquired as displayed in Fig. 10.

$$\begin{array}{l} \left[t_{p},t_{q}\right]|C={\left[\begin{array}{cccccccccccc} 72 & 50 & 40 & 42 & 68 & 78 & 74 & 64 & 46 & 56 & 70 & 80\\ 114 & 142 & 156 & 152 & 122 & 104 & 110 & 128 & 148 & 134 & 116 & 102 \end{array}\right]}^{T}\\ {}[t_{g},t_{h}]|R={\left[\begin{array}{c} 52\\ 146 \end{array}\;\begin{array}{c} \text{76}\\ \text{102} \end{array}\;\begin{array}{c} \text{82}\\ \text{98} \end{array}\right]}^{T} \end{array}$$

Given Formula (4) and set *k*_1_ = *k*_2_ = 1, the value of *σ*_*ij*_(*i* = 1, 2, …12, *j* = 1, 2, 3) is calculated as

$$\sigma_{ij}=\left[\begin{array}{ccc} 0 & 0.3062 & 0.2250\\ 0.5102 & 0.1361 & 0.2656\\ 0.9051 & 0.1461 & 0.3129\\ 0.1113 & 0.1418 & 0.2984\\ 0 & 0.1930 & 0.2277\\ 0 & 0.0772 & 0.3359\\ 0 & 0.5503 & 0.2344\\ 0 & 0.1603 & 0.2352\\ 0.1775 & 0.1398 & 0.2856\\ 0 & 0.1358 & 0.2416\\ 0 & 0.2258 & 0.2161\\ 0 & 0.0780 & 0.5781 \end{array}\right]$$

**Fig 11:**
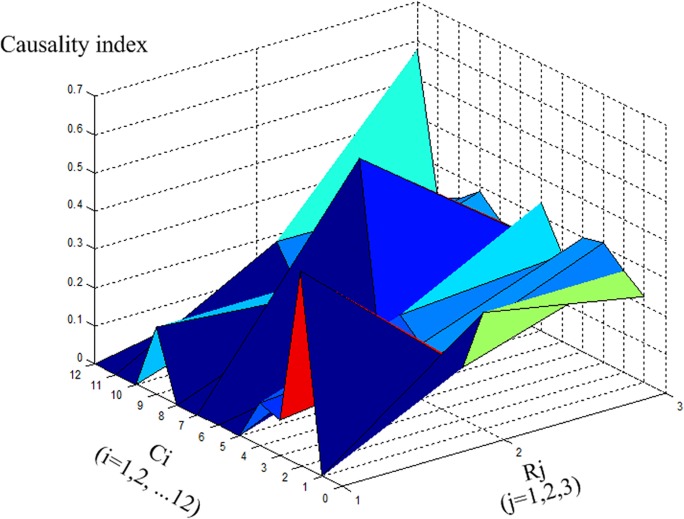
Causality index map.

Fig. 11 shows the 3D surface curve for the causality index values. A peak curve indicates strong causality, whereas a valley curve denotes weak causality. The causality distribution map is thus demonstrated quantitatively.

### System fault location

**Fig 12:**
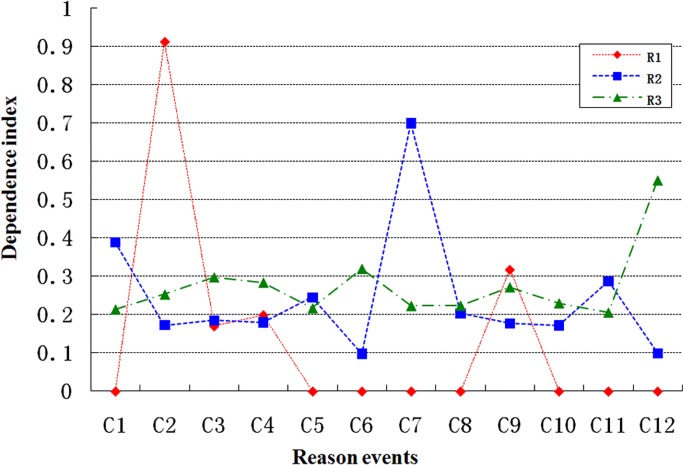
Dependence index map.

Initially, the dependence connection strength is set as *W*_*d*_(*C*_*i*_, *R*_*j*_) = 1/12 using Formula (5). The calculation result of *DEP*_*i*_(*R*_*j*_) (*i* = *1*, *2*, *…12*) is depicted in Fig. 12.

We simply aim to determine the greatest potential locations of system fault; thus, we can ignore the reason events with very low dependence index values. In line with this objective, we developed a threshold dependence index value to simplify the comparison process.

The threshold dependence index value for the dependence index in system fault R1 is set to 0.15. The dependence indices that exceed this threshold are *DEP*_2_(*R*_1_), *DEP*_3_(*R*_1_), *DEP*_4_(*R*_1_), and *DEP*_9_(*R*_1_). These indices can be arranged in descending order as follows:

$$DEP_{2}(R_{1})>DEP_{9}(R_{1})>DEP_{4}(R_{1})>DEP_{3}(R_{1})>0.15$$

This relationship indicates the dependence levels of *R*_1_ on *C*_2_, *C*_9_, *C*_4_, and *C*_*3*_. *C*_*2*_ is the most likely reason event for system fault 1. Thus, the sensor location is *C*_2_. The other potential locations of system fault 1 are *C*_9_, *C*_4_, and *C*_3_.

If the threshold value of the dependence index is set to 0.2, then the potential locations of system fault 2 are *C*_7_, *C*_1_, *C*_11_, *C*_5_, and *C*_8_, given *DEP*_7_(*R*_2_) > *DEP*_1_(*R*_2_) > *DEP*_11_(*R*_2_) > *DEP*_5_(*R*_2_) > *DEP*_8_(*R*_2_) > 0.2.

Similarly, the potential locations of system fault 3 are *C*_12_, *C*_6_, *C*_3_, *C*_9_, and *C*_2_ if the threshold value for the dependence index is set to 0.25, given

$$DEP_{12}(R_{3})>DEP_{6}(R_{3})>DEP_{3}(R_{3})>DEP_{9}(R_{3})>DEP_{2}(R_{3})>0.25.$$

The experimental results are consistent with the intuitive spatial analysis results. A precise mathematical structural model is unnecessary to quantitatively calculate the causal relationships between the monitoring processes and the results and locate the system fault.

### Update of the connection strength

**Fig 13:**
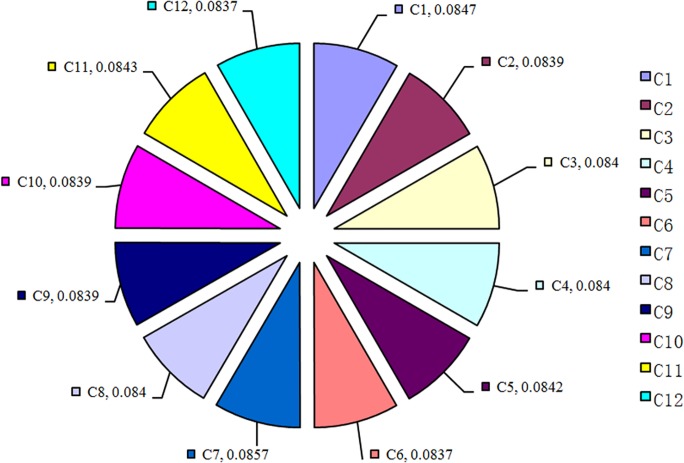
Dependence connection strength between R2 and Ci (i = 1, 2, …12).

Once *DEP*_*i*_(*R*_*j*_) (*i* = 1, 2, …*m*, *j* = 1, 2, …*n*) is computed, the new value of *W*_*d*_(*C*_*i*_, *R*_*j*_) can be regulated dynamically using Formula (6). An example of the new value of *W*_*d*_(*C*_*i*_, *R*_2_)(*i* = 1, 2, …12) is provided in Fig. 13.

The data in this figure show that *W*_*d*_(*C*_7_, *R*_2_) > *W*_*d*_ (*C*_1_, *R*_2_) > *W*_*d*_ (*C*_11_, *R*_2_)> ……, which is similar to the data trend presented in Fig. 12. If the value of the current dependence index is high, then dependence connection strength is enhanced to improve subsequent probabilistic reinforcement.

### Comparison experiment based on the benchmark dataset

The fault identification procedure is carried out in the Los Alamos National Laboratory of the United States. Specifically, the factor analysis, principal component analysis, and Mahalanobis distance methods are adopted to analyze the faults in a three-story building structure.

To demonstrate the validity of the presented model, the model is applied to a three-story building structure. Algorithms are compared using experimental benchmark datasets.

### Introduction of the three-story building structure

**Fig 14:**
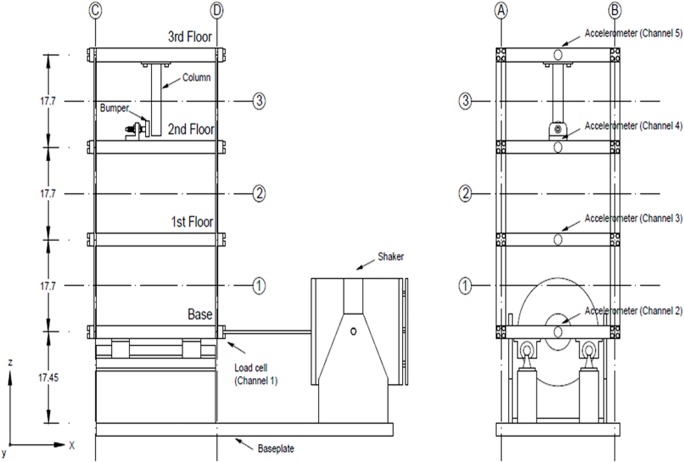
Three-story building structure.

As shown in Fig. 14, the structure consists of aluminum columns and plates assembled using bolted joints. A center column is suspended from the top floor. This column can be used to simulate fault by inducing nonlinear behavior when it contacts a bumper mounted on the next floor. In the context of SHM, this source of fault is intended to simulate fatigue cracks that can open and close or loose connections that can rattle under dynamic loading. An electrodynamic shaker provides a lateral excitation to the base floor along the centerline of the structure. A load cell (Channel 1) with a nominal sensitivity of 2.2 mV/N was attached at the end of a stinger to measure the input force from the shaker to the structure. Four accelerometers (Channel 2–5) with nominal sensitivities of 1000 mV/g were attached at the centerline of each floor on the opposite side from the excitation source to measure the system response[38].

### Application of the model based on the benchmark dataset

The structural fault in the three-story building structure is caused by shaker shock and by the collision between the bumper and the column. Channel 1 is located near the shaker, and Channel 4 is positioned on the bumper layer. Thus, Channels 1 and 4 are considered reason event set C = {C_1_, C_2_}. Channels 2, 3, and 5 are regarded as result event set R = {R_1_, R_2_, R_3_} in our experiment. Among these channels, Channel 2 is nearest to Channel 1 and is farthest from Channel 4. Thus, the fault monitored by Channel 2 (R_1_) is mainly attributed to Channel 1 (C_1_). This fault originates from shaker shock. Channel 3 is located between Channels 1 and 4. Thus, the fault monitored by Channel 3 (R_2_) is primarily caused by the comprehensive influences of Channels 1 (C_1_) and 4 (C_2_). Channel 5 is nearest to Channel 4 and is farthest from Channel 1. Therefore, the fault monitored by Channel 5 (R_3_) is mainly ascribed to Channel 4 (C_2_). This fault is in turn generated by the collision between the bumper and the column.

In the experiment, 500 sample points at each channel state are collected from the benchmark dataset. These points are regarded as the raw experimental data. Given that state 1 represents the baseline condition, the raw data of state 1 are subtracted from the raw data of states 2–17 to acquire the preprocessing data.

**Fig 15:**
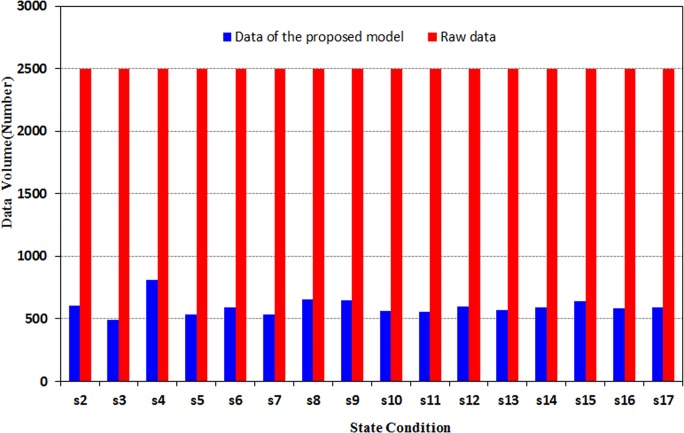
Contrastive chart of data volume.

**Fig 16:**
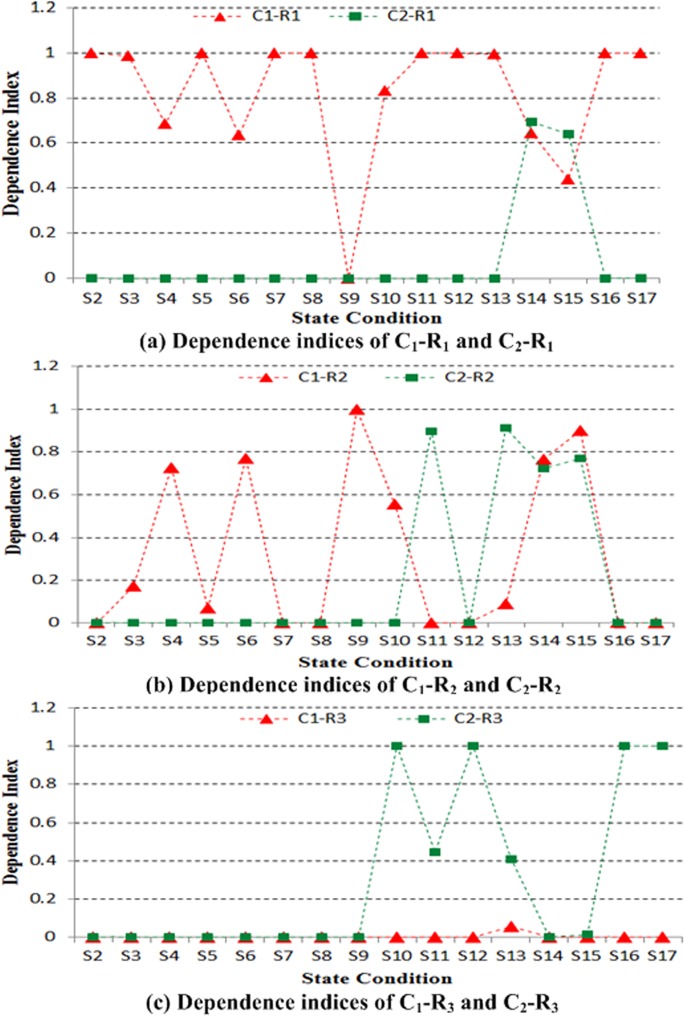
Dependence indices between the result and reason events.

**Fig 17:**
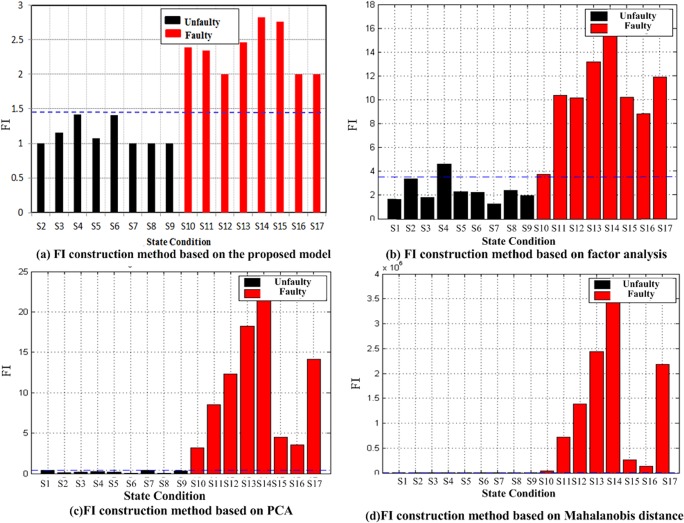
Comparison of algorithms for the fault indicator.

The proposed model is then used to preprocess the data. The volume of the results data is compared with that of the raw data, as shown in Fig. 15. S1–S17 in Figs. 15–17 represent states 1–17, respectively.

Fig. 15 indicates that the volume of data has been compressed to 25% of the raw data.

Safety thresholds are first set for each channel in the SIS and STS stages of the proposed model. Subsequently, the measured data are matched against the safety thresholds. If the data in the channel exceed the safety threshold, then the time value of the monitored data is recorded as the onset time of event *i* (reason or result event). If the absolute value of the measured data remains greater than the safety threshold, then the time value of the monitored data is recorded as the end time of event *i*. Table 1 depicts a time set of events as per the proposed model.

**Table 1:** Time set of events as per the proposed Model.

State	Channel1(C_1_)	Channel4(C_2_)	Channel2(R_1_)	Channel3(R_2_)	Channel5(R_3_)
**state2**	[1,12]	[35,38]	[12,15]	[32,34]	[20,23]
**state3**	[1,11]	[20,27]	[1,54]	[5,7]	[75,78]
**state4**	[1,5]	[7,9]	[1.1,4]	[1.1,3]	[88,91]
**state5**	[1,5]	[4,6]	[1.1,2]	[2,9]	[28,31]
**state6**	[1,9]	[102,108]	[1,12]	[1,3]	[105,108]
**state7**	[45,48]	[1,4]	[45.1,47]	[8,14]	[21,62]
**state8**	[1,3]	[49,52]	[1.1,3]	[45,47]	[80,81]
**state9**	[1,5]	[20,22]	[11,13]	[1.1,2]	[0,0]
**state10**	[1,39]	[9,11]	[1,2]	[2,3]	[9,23]
**state11**	[1,13]	[20,22.5]	[1.1,13.1]	[18,21]	[22,25]
**state12**	[1,11]	[35,39.1]	[7,11.1]	[12,13]	[39,40]
**state13**	[1,10]	[4,6]	[1.1,2]	[2,5]	[6,8]
**state14**	[1,10]	[1,3]	[1.1,7]	[1.1,3.1]	[0,0]
**state15**	[1,7]	[2,11]	[2.1,7.1]	[2.1,4]	[8,13]
**state16**	[1,15]	[25,27]	[1.1,16]	[18,21]	[22,25.1]
**state17**	[1,9]	[22,25]	[1.1,19]	[18,20]	[22.1,24]

Based on the aforementioned time set, the dependence index between the reason and result events can be obtained at different states as presented in Table 2.

**Table 2:** Dependence index between the reason and result events at different states.

State	C_1_-R_1_	C_2_-R_1_	C_1_-R_2_	C_2_-R_2_	C_1_-R_3_	C_2_-R_3_	FI
**state2**	1	0	0	0	0	0	1
**state 3**	0.985	0	0.1723	0	0	0	1.1573
**state 4**	0.6866	0	0.727	0	0	0	1.4136
**state 5**	0.9974	0	0.0714	0	0	0	1.0688
**state 6**	0.6368	0	0.771	0	0	0	1.4078
**state 7**	1	0	0	0	0	0	1
**state 8**	1	0	0	0	0	0	1
**state 9**	0	0	1	0	0	0	1
**state 10**	0.8311	0	0.5561	0	0.0041	1	2.3913
**state 11**	1	0	0	0.8944	0	0.4472	2.3416
**state 12**	1	0	0	0	0	1	2
**state 13**	0.9944	0	0.0895	0.9119	0.0569	0.4104	2.4631
**state 14**	0.6427	0.6925	0.7661	0.7214	0	0	2.8227
**state 15**	0.4383	0.6389	0.8988	0.7691	0	0.0177	2.7628
**state 16**	1	0	0	0	0	1	2
**state 17**	1	0	0	0	0	1	2

Fig. 16(A)–16(C) exhibit the dependence indices between the result and reason events. The structural fault location can then be analyzed.

Prior to state S10, bumper—column collision does not generate faults, as displayed in Fig. 16(A). The dependence indices of C_1_-R_1_ almost exceed 0.6, and all of the dependence indices of C_2_-R_1_ are 0. This phenomenon suggests that the fault monitored by Channel 2 is influenced by C_1_ (shaker) alone. Following the action of the bumper from the state S10, all of the dependence indices of C_1_-R_1_ are greater than those of C_4_-R_1_. This finding indicates that the fault monitored by Channel 2 is mainly caused by C_1_ (shaker).

Under the same bumper operation condition, Fig. 16(B) suggests that the dependence indices of C_1_-R_2_ are almost similar to those of C_2_-R_2_. It also indicates that the fault monitored by Channel 3 is primarily induced by C_1_ (shaker) and C_4_ (bumper).

Fig. 16(C) also reveals that the dependence indices of C_2_-R_3_ are greater than those of C_1_-R_3_ following bumper operation from state S10. This finding suggests that the fault monitored by Channel 5 is mainly attributed to C_4_ (bumper).

In summary, Fig. 16(A)–16(C) indicate that the fault monitored by Channel 2 is primarily caused by shaker shock; that the fault monitored by Channel 3 is mainly induced by shaker shock and bumper—column collision; and that the fault monitored by Channel 5 is primarily attributed to the collision between the bumper and the column. The experimental result is consistent with the structural fault analysis conducted previously.

### Comparison of algorithms

Faults were analyzed using the factor analysis[39], principal component analysis[38], and Mahalanobis distance [40]methods in the Los Alamos National Laboratory of the United States. Mahalanobis distance is a weighted Euclidean distance that considers the links among various characteristics and can calculate the similarity between two sample sets, whereas structural fault identification can be regarded as a process that evaluates the similarity between the measurement datasets and the normal dataset. The experimental data in this article are derived from multiple channels and are in multiple states. Factor analysis and PCA methods can reduce dimensionality, and this feature can help simplify the problem to be addressed in the experiment. The benchmark dataset originates from Los Alamos National Laboratory of the United States. Researchers at the laboratory have applied factor analysis, PCA, and Mahalanobis distance methods to analyze faults in a three-story building structure. Therefore, the proposed method is compared objectively with the aforementioned methods. The calculation of the fault analysis consumed most of the time and space during fault location; thus, the algorithm of the proposed model was compared with three other typical algorithms. The comparison is based on the experimental data acquired from channels 1–5 at five different locations. In the experiment conducted by the Los Alamos National Laboratory of the United States, shaker shock represents the environmental changes to the structure, and the structure is considered unfaulty. Bumper collision is regarded as the source of structural faults, and the structure is regarded as faulty. The data acquired from channels 1–5, which are located at different layers, are adopted to determine whether or not the overall state of a three-story building structure is faulty. During the experiment, shaker shock alone is introduced into S1–S9. Subsequently, the algorithms are processed to assess whether or not the structure remains in the desired unfaulty state. By contrast, both shaker shock and bumper collision are introduced into S10–S17. Then, the algorithms are then processed to evaluate whether or not the structure remains in the desired faulty state.

In the proposed experimental method, the sum of the dependence indices with similar states is regarded as the fault indicator (FI) of the presented model. Part of the preprocessed data is used in model training to obtain an FI threshold value of 1.5 for this experiment. Then, the test data are inputted into the trained model to determine the corresponding FI value. If the FI value is higher than the threshold, then the structure is considered faulty. The results are shown in Fig. 17(A).

The FI construction method based on factor analysis operates as follows: Raw data are inputted into an autoregressive (AR) model to acquire AR parameters. These data are then regarded as fault-sensitive features. Subsequently, the factor analysis model is used to calculate the factor cores for the fault-sensitive feature vectors under a normal structural condition. The fault indicator is determined through these cores. A simple FI threshold is then generated after model training. The fault identification results are shown in Fig. 17(B).

The FI construction method based on PCA operates as follows: Raw data are inputted into an AR model, and the root mean square (RMS) errors of this model are considered fault-sensitive features. A machine learning algorithm based on PCA is used to obtain a fault indicator that is invariant for feature vectors under a normal structural condition. This indicator is enhanced when the feature vectors originate from the fault structural condition. The fault identification results are depicted in Fig. 17(C).

The FI construction method based on Mahalanobis distance operates as follows: Raw data are inputted into an AR model, and its parameters are used as fault-sensitive features. The Mahalanobis distance model is then trained by fault-sensitive feature vectors obtained under a normal structural condition. The FI is determined by calculating the Mahalanobis distance from these fault-sensitive feature vectors to their mean vector. The fault identification results are presented in Fig. 17(D).

**Fig 18:**
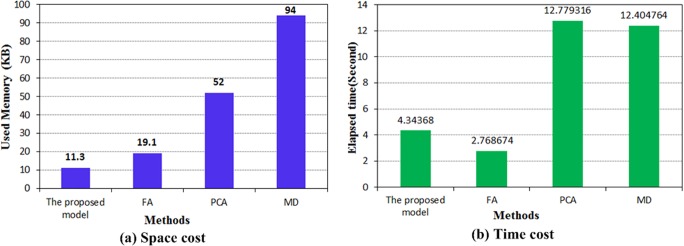
Comparison of algorithms for space and time cost.

Fig. 18 depicts the comparison of space and time consumption. Fig. 17 indicates that the method incorporating factor analysis includes an FI construction technique based on the factors for all of the states except S4. Thus, the two groups can be separated by a simple threshold. Although this method is faster than that of the proposed model, its space storage cost is greater. The methods that use principal component analysis and Mahalanobis distance generate FI construction techniques to separate the two groups according to a simple threshold. These techniques identify faults more effectively than the proposed model does. However, they require much sampling data to train the model. Thus, they incur higher space and time costs than the proposed model does. Based on this comparison, the proposed model can effectively identify structural faults at low space and time costs.

## Conclusion

A SHM system uses numerous sensors. However, this system is plagued by the two challenges of massive data storage pressure and structural fault location. To address these concerns, the current research proposed the bio-inspired memory model embedded with causality reasoning function for structural fault location. First, the model filters much normal data. Thus, only the critical data that reflect structural change are recorded in the three memory areas. Therefore, the storage pressure on the SHM system is lowered. Second, the model is embedded with a quantitative causal reasoning function that includes two causal indices, namely, causality and dependence. The causality index indicates the causality strengths of the reason and result events. This index is associated with the function of causal knowledge discovery. The dependence index indicates the dependence levels of a result event on the reason events. This index is used to reason out the sensor locations of the structural fault. Experiments demonstrate that the proposed model can effectively identify structural faults at low space and time costs.
